# The effect of basic medical insurance on the changes of primary care seeking behavior: An application of hierarchical age-period-cohort analysis

**DOI:** 10.3389/fpubh.2022.929896

**Published:** 2022-08-03

**Authors:** Na Cao, Xuyang Li, Junfeng Jiang, Wenyan Xu

**Affiliations:** ^1^School of Public Health, Wuhan University, Wuhan, China; ^2^School of Sociology, Central China Normal University, Wuhan, China; ^3^Jiangxi Provincial People's Hospital, The First Affiliated Hospital of Nanchang Medical College, Nanchang, China

**Keywords:** basic medical insurance, HAPC-CCREM, China, primary care, seeking behavior

## Abstract

In order to encourage residents to go to primary care facilities, China has set up differentiated basic medical insurance reimbursement ratios. The study aims to use the dynamic point of view of longitudinal data to examine the changes in the impact of basic medical insurance on primary care. The data for this study comes from the Chinese Family Panel Study (CFPS) in 2010, 2012, 2014, 2016, and 2018. We adopted Hierarchal Age-period-cohort-Cross-Classified Random Effects Models (HAPC-CCREM) to examine the changes in the impact of basic medical insurance on primary care. Compared with non-insured groups, participants of the New Rural Cooperative Medical System (coefficient = 0.730) have a relatively high incidence of primary care seeks, while Urban Residents' Basic Medical Insurance (coefficient = −0.482) and Urban Employees' Basic Medical Insurance (coefficient = −0.663) are lower, respectively. Age, period over time and cohort have a more obvious moderating effect on primary care seeks. The study of primary care behavior is an important direction for the construction of a hierarchical medical system. As basic medical insurance is the source of power for the hierarchical medical system, we can provide certain direction for policy formulation on the changes of basic medical insurance in primary care behavior.

## Introduction

In China, the first diagnosis at the primary level guides patients to seek health service at the primary care facilities, which can optimize the allocation of resources and standardize the order of medical treatment. However, there has been a tendency for people to pursue higher-level hospitalization services, with the rate of admissions to primary care facilities decreasing from 38.4% to 26.4% between 2009 and 2015 (1). Only 55.19% of the patients visited primary health care in 2018, in China (2). In order to guide patients to the primary for medical treatment, China has set a differentiated basic medical insurance reimbursement ratio (3). China has developed three key basic medical insurance programs for different groups. Among them, the Urban Residents' Basic Medical Insurance (URBMI) established in 1998 is for urban employees, the New Rural Cooperative Medical System (NRCMS) initiated in 2003 is for rural residents, and the Urban Employees' Basic Medical Insurance (UEBMI) established in 2007 is for urban residents who are not covered by the URBMI, such as the elderly without pension, children and college students. The UEBMI's participants are higher-paid and higher-guaranteed, the premiums are divided amongst employers and employees by a rate set. The NRCMS and URBMI's participants are lower-paid and lower-guaranteed, the Central and provincial governments subsidize most of the premiums, and the participants only need to pay a small part. What's more, there are certain differences in the reimbursement ratios of the these three basic medical insurance types, and the reimbursement ratios of primary medical facilities are higher than that of high-level hospitals (4).

### Definitions of age, period, and cohort effects

Age, period, and cohort effects all refer to some type of time-related variation in the phenomena of interest, yet they carry distinct substantive meanings (5). The age effect is defined as the variation between different age groups caused by physiological changes, accumulation of social experience and changes in role status. Berhanu's research results show that nearly half of medical costs occur in advanced age. For 85-year-old survivors, more than one-third of their lifetime expenses will be accumulated in the remaining years (6). The relatively low cost of primary care will make the elderly more willing to choose primary care. However, when the age increases to a certain level, primary medical facilities can no longer meet their health needs. At this time, these elderly people can only go to the hospital to bypass the primary medical institutions.

Period effects are defined as variation over time periods or calendar years that influence all age groups simultaneously. Period effects include a series of complex historical events and environmental factors (5). Shift in social, cultural, economic, or physical environments may in turn induce similar changes in the lives of all people at a given point in time. Past empirical studies have shown that the proportion of primary care in 2013 was lower than that in 2011 (7). In 2015, the Chinese government issued the “Guiding Opinions of the General Office of the State Council on Pushing Forward the Building of the Hierarchical Medical System,” which marked the beginning of the formal implementation of the construction of a hierarchical diagnosis system in our country and provides more possibilities for primary medical treatment. The China Health Service Survey in 2018 mentioned that the accessibility of urban and rural health services has been further improved, showing the effect of implementing a hierarchical diagnosis system. However, the choice of medical service institutions is a habitual behavior, which is difficult to be changed in a short period of time.

Cohort effects are defined as variation between groups of people who experience an initial event such as birth or marriage in the same year or years. Birth cohorts are the most commonly used unit of analysis in demographic and aging research. Since the founding of China (1949 to 1970s), China's community health services have developed rapidly. Rural areas have established a primary care and health service system with barefoot doctors who have no formal medical training, still hold an agricultural household registration, and in some cases “semi-agricultural and half-medicine” as the backbone, and cities have established a large number of community health service stations (8). The medical reform in 1985 made the allocation of health resources appear to be concentrated. The development trend of globalization has accelerated the construction of large hospitals, but the primary care have insufficient business volume. The new medical reform in 2009 proposed to vigorously develop community health services. Two rounds of medical reform have affected China's health resource allocation pattern. These historical events or the process of social change not only affected the attitudes or behaviors of the population's primary care at different time points, but also had obvious differences in the impact on different birth cohorts at the same time point. Although we have not retrieved relevant studies at present, different birth cohort groups may have different primary care due to their specific experience background, which is also in line with the thinking of life course theory.

### The necessity to explore of the impact of medical insurance on primary care seeking behavior

In the past, a great number of papers found that the basic medical insurance system will have an effect on the primary care. However, they only pay attention to whether they are insured or a certain type of basic medical insurance, ignoring the differences between different basic medical insurances (9). In addition, the attention of basic medical insurance to primary care was limited to a static perspective. In fact, the change in personal attitudes or behaviors may be affected by the life process from birth to death, with each birth cohort going through a specific life stage. With the passage of age, period, and birth cohort, the trajectory of changes in the behavior of the primary care may also change. Therefore, is there any heterogeneity in the impact of different basic medical insurance types on primary diagnosis behavior at the primary level? What is the impact of age, period, and cohort on primary medical behavior? In the process of age-period-cohort, has the effect of different basic medical insurance types changed? For these questions, the answers have not been found in previous studies.

### Purpose of this study

The unbalanced distribution of health resources and the ineffective utilization of primary medical facilities are the core issues that restrict China's medical and health reform. This paper is a response to this social hot spot. In addition, this paper also has the following innovations. First, this study is based on the nationwide residents' data to test the implementation of primary medical diagnosis and treatment, and to understand the current status of primary medical diagnosis and treatment of residents as a whole. Second, we examine the guiding effect of the basic medical insurance system on the selection behavior of resident institutions and consider the differences between the three basic medical insurance systems. Third, we consider the impact of basic medical insurance on primary care under the observation framework of age, period, and cohort. This is the first time that the impact of basic changes in medical insurance on primary care has been found. We re-examined the relationship between basic medical insurance types and primary care-seeking behavior in the transformation of the personal life course, changes in the times and the group experience of birth cohorts. Therefore, this study aims to solve the following two problems:

a. Will basic medical insurance participation, age, period, and cohort affect the behavior of seeking primary care?
b. Does the impact of basic medical insurance participation on the behavior of seeking primary care vary with age, period, and cohort?

## Methods

### Data source and study sample

This study used a large sample derived from five waves of the CFPS survey (2010–2018), which collect individual-, family-, and community-level longitudinal data. The baseline survey was conducted in 2010, using multistage probability proportional to size sampling. Counties or administrative equivalents were drawn from 25 selected provinces, and then communities were drawn from selected counties or administrative equivalents. The socioeconomic level was used as an indicator of implicit stratification at these two stages. At the third stage, 25 households were randomly drawn from each sampled community based on the onsite sampling frame, and members of every household were asked to participate in the survey. The baseline sample represents 95% of the Chinese population, with an approximate response rate of 79%. Details of the sample design have been described in other studies and on the website (10). The survey aims to conduct a comprehensive and in-depth investigation of family relationships and family member information, establish a family structure network that can clearly locate the relationship between family members, and hope to be able to provide people with a broader vision for understanding and studying Chinese society. The baseline survey was completed in 2010, and the follow-up surveys were completed in 2012, 2014, 2016, and 2018, respectively. There were 32,918 samples in 2010, 34,937 samples in 2012, 34,888 in 2014, 28,450 samples in 2016, 31,683 samples in 2018 initially. Participants who were younger than 18 years old (3,749 samples) were excluded from the analysis, and deleted the samples with missing values or outliers based on our study, with a final sample of 85,316 participants.

### Variables and measures

#### Dependent variable–primary care

Primary care was measured using the question “where do you usually go to seek health services when you sick?” with answer options of 1 = “community health service center/township health center,” 2=“community health service station/village clinic,” 3 = “clinic,” 4 = “general hospitals,” and 5 = “specialty hospitals.” Considering whether residents go to primary care facilities is to measure the implementation effects of major measures such as China's deepening reform planning and implementation scheme as of medical and health (11, 12). According to national standards, community health service center/township health center/community health service station/village clinic are defined as primary medical facilities. Therefore, we set the primary treatment as a binary variable, and further categorized the answer options into 1 = “Residents who choose primary care” (including 1 = “community health service center/township health center,” 2 = “community health service station/village clinic,” 3 = “clinic”), 0 = “Residents who do not choose primary care” (including 4 = “general hospitals,” and 5 = “specialty hospitals”) (7).

#### Independent variables–medical insurance

Medical insurance was measured using the question “What kind of medical insurance do you have?” with answer options of 1 = “Free medical treatment,” 2 = “Urban Residents' Basic Medical Insurance,” 3 = “Urban Employees' Basic Medical Insurance,” 4 = “Supplementary medical insurance,” 5 = “New Rural Cooperative Medical System,” and 6 = “not participating in any basic medical insurance.” Because Free medical treatment and Supplementary medical insurance do not belong to the scope of China's basic medical insurance, so we further categorized the variable into 1 = “NRCMS,” 2 = “URBMI,” 3 = “UEBMI”, and 0 = “not participating in any basic medical insurance” (13).

#### Age, period, and cohort variables

This study focused on the three dimensions of age, period, and cohort. The age variable is the actual age of the respondent at the time of the interview, which is a continuous variable and was placed on the first level. The cohort and period were placed on the second level. The period was measured by the year of the survey. As mentioned above, it is 2010 and 2012, 2014, 2016, 2018. The cohort was measured by the year of birth, and 5 years was an interval, from the pre 1935 group, the 1940 group, … until the 1995 group, a total of 14 group.

#### Covariates

Gender (male, female), marital status (unmarried, married, divorced/widowed), income (in years, continuous variables), education (primary school, middle school, high school, associate degree or above), self-assessed health status (unhealth, health), and chronic disease (no, yes) were included as covariates in the study. The standard deviation of income was relatively large, and the degree of dispersion was relatively high. The logarithmic transformation was used to correct the non-normality of the data.

### Statistical analysis

Descriptive analyses (mean, standard deviation, frequency, percentage) were used to present the sample characteristics. The prevalence of primary care was presented by period and cohort.

As we discussed in the introduction section, the prevalence of seeking primary care behaviors at specific years consists of three time-related components: age, time period and birth cohort. Thus, it is biased when presenting the prevalence without considering the age and cohort effect. However, due to the complete collinearity of the three time-related components (e.g., age = period-cohort), conventional statistical models were unable to address the issue. In recent years, Hierarchal Age-Period-Cohort Cross Classified Random Effects Models (HAPC-CCREM) was developed by Yang Yang and colleagues to solve the collinearity issue by breaking the natural collinearity among the three through stratifying the influence of age, period and cohort on the dependent variable (14).In a repeated cross-sectional survey, the HAPC model is an effective apparatus to estimate the effects of age, period, cohort (15).

In this study, age was placed on the first level of individuals (i.e., fixed effect) while period and cohort were placed on the second macro-level (i.e., random effect). The following regression strategy was employed: we used a basic HAPC-CCREM to explore the relationship between basic medical insurance, age, period, cohort and Primary care-seeking behavior. In order to examine whether the impact of basic medical insurance participation on the behavior of seeking primary care varies with age, period, and cohort, then we introduced the interaction items of basic medical insurance and age, basic medical insurance and period, basic medical insurance and cohort on new models based on above HAPC-CCREM. More information details can be seen in follow informative equations.

First, a basic model was used to investigate the impacts of basic medical insurance, age, period, and cohort on primary medical facilities (Equations 1–2).

Level 1:


(1) $$\begin{array}{l} Y_{ijk}=\beta_{0ijk}+\beta_{1}Age_{ijk}+\beta_{2}Age_{ijk}^{2}+\beta_{3}Sex_{ijk}+\beta_{4}Income_{ijk}\\ +\beta_{4}Education_{ijk}+\beta_{5}SRH_{ijk}+\beta_{6}Chronic_{ijk}+\beta_{7}Insurance_{ijk}\\ +e_{ijk}\;,\;e_{ijk}\sim N\left(0,\delta^{2}\right) \end{array}$$

Level 2:


(2) $${\text{β}}_{\text{0jk}}{\text{=γ}}_{\text{0}}{\text{+μ}}_{\text{0j}}{\text{+ν}}_{\text{ok}}\text{ , }{\text{μ}}_{\text{0j}}\text{\textasciitilde{} N}\left({\text{0,ε}}_{\text{u}}\right)\text{ , }{\text{ν}}_{\text{ok}}\text{\textasciitilde{} N}\left({\text{0,ε}}_{\text{υ}}\right)$$

Secondly, we investigated the interaction between the age effect and the medical insurance on the medical treatment behaviors (Equations 3–4).

Level 1:


(3) $$\begin{array}{l} {\text{Y}}_{\text{ijk}}=\beta_{0ijk}+\beta_{1}Age_{ijk}+\beta_{2}Age_{ijk}^{2}+\beta_{3}Sex_{ijk}+\beta_{4}Income_{ijk}\\ +\beta_{4}Education_{ijk}+\beta_{5}SRH_{ijk}+\beta_{6}Chronic_{ijk}\\ +\beta_{7}Insurance_{ijk}+\beta_{8}Insurance^{*}Age_{ijk}\\ +e_{ijk}\;,\;e_{ijk}\sim N\left(0,\delta^{2}\right) \end{array}$$

Level 2:


(4) $${\text{β}}_{\text{0jk}}{\text{=γ}}_{\text{0}}{\text{+μ}}_{\text{0j}}{\text{+ν}}_{\text{ok}}\text{ , }{\text{μ}}_{\text{0j}}\text{\textasciitilde{} N}\left({\text{0,ε}}_{\text{u}}\right)\text{ , }{\text{ν}}_{\text{ok}}\text{\textasciitilde{} N}\left({\text{0,ε}}_{\text{υ}}\right)$$

Thirdly, the interaction between the period effect and medical insurance (Equations 5–7).

Level 1:


(5) $$\begin{array}{l} Y_{ijk}=\beta_{0ijk}+\beta_{1}Age_{ijk}+\beta_{2}Age_{ijk}^{2}+\beta_{3}Sex_{ijk}+\beta_{4}Income_{ijk}\\ +\beta_{4}Education_{ijk}+\beta_{5}SRH_{ijk}+\beta_{6}Chronic_{ijk}\\ +\beta_{7}Insurance_{ijk}+e_{ijk}\;,\;e_{ijk}\sim N\left(0,\delta^{2}\right) \end{array}$$

Level 2:


(6) $${\text{β}}_{\text{0jk}}{\text{=γ}}_{\text{0}}{\text{+μ}}_{\text{0j}}{\text{+ν}}_{\text{ok}}\text{ , }{\text{μ}}_{\text{0j}}\text{\textasciitilde{} N}\left({\text{0,ε}}_{\text{u}}\right)\text{ , }{\text{ν}}_{\text{ok}}\text{\textasciitilde{} N}\left({\text{0,ε}}_{\text{υ}}\right)$$

(7) $${\text{β}}_{\text{7j}}{\text{=γ}}_{\text{0}}{\text{+μ}}_{\text{0j}}\text{ , }{\text{μ}}_{\text{0j}}\text{\textasciitilde{} N}\left({\text{0,ε}}_{\text{u}}\right)$$

Finally, we investigated the interaction between the cohort effect and medical insurance as shown Equations 8–10.

Level 1:


(8) $$\begin{array}{l} Y_{ijk}=\beta_{0ijk}+\beta_{1}Age_{ijk}+\beta_{2}Age_{ijk}^{2}+\beta_{3}Sex_{ijk}+\beta_{4}Income_{ijk}\\ +\beta_{4}Education_{ijk}+\beta_{5}SRH_{ijk}+\beta_{6}Chronic_{ijk}\\ +\beta_{7}Insurance_{ijk}+e_{ijk}\;,\;e_{ijk}\sim N\left(0,\delta^{2}\right) \end{array}$$

Level 2:


(9) $${\text{β}}_{\text{0jk}}{\text{=γ}}_{\text{0}}{\text{+μ}}_{\text{0j}}{\text{+ν}}_{\text{ok}}\text{ , }{\text{μ}}_{\text{0j}}\text{\textasciitilde{} N}\left({\text{0,ε}}_{\text{u}}\right)\text{ , }{\text{ν}}_{\text{ok}}\text{\textasciitilde{} N}\left({\text{0,ε}}_{\text{υ}}\right)$$

(10) $${\text{β}}_{\text{7k}}{\text{=γ}}_{\text{0}}{\text{+ν}}_{\text{ok}}\text{ , }{\text{ν}}_{\text{ok}}\text{\textasciitilde{} N}\left({\text{0,ε}}_{\text{υ}}\right)$$

In the above models, primary care is a binary variable, so we adopt the logit link function in all above models. The REML estimation method was used for parameter estimation (16). All models were implemented using PROC GLIMMIX in SAS (version 9.4, Cary NC, USA).

## Results

### Characteristics of the study sample

Results in Additional file 1: Supplementary Table 1 show that among a total sample of 85,316, 50.67% were male, 83.63% married, 10.02% unmarried, 49.06% had received primary school education, and their mean income yearly was 120,889 (SD). Of the total sample, 36.38% perceived them as healthy, and 13.87% had at least one chronic disease. The participation rate of NRCMS, URBMI, UEBMI was 69.83%, 7.09%, 12.97%, respectively. And only 10.11% of the samples did not participate in any insurance type, and 89.89% of the samples had received primary care.

Results in Additional file 2: Supplementary Table 2 present the period and cohort distribution of primary care seeking behavior. It is found that the prevalence of primary care seeking behavior was higher in period 2010, and in more recent periods, earlier cohorts were more likely to go to primary medical facilities for health care seeking.

### Age, period, and cohort effect of seeking primary care

Results in Additional file 3: Supplementary Figure 1 reveal that the age effect of seeking primary care increased consistently with age. The probability of seeking primary care declined from 2010 to 2016 but slightly rebounded in 2018. The cohort effect on the probability of seeking primary care was weak, and only the cohort born in 1981–1985 had a significant lower probability of seeking primary care. More detail can be seen in Table 1.

**Table 1:** The effect of basic medical insurance participation, age, period, and cohort on behavior of seeking primary care.

	**Model 1 (*****N*** = **85316)**					**Model 2 (*****N*** = **85316)**					**Model 3 (*****N*** = **85316)**				
	**Estimate**	**Standard error**	**OR**	**95%CI**		**Estimate**	**Standard error**	**OR**	**95%CI**		**Estimate**	**Standard error**	**OR**	**95%CI**	
				**Lower**	**Upper**				**Lower**	**Upper**				**Lower**	**Upper**
**Fixed effect**												
Intercept	0.685***	0.039	1.983	1.836	2.141	0.904*	0.418	2.469	1.001	6.094	0.894*	0.283	2.444	1.116	5.354
Age	0.010**	0.004	1.011	1.004	1.017	−0.054***	0.009	0.957	0.940	0.974	0.012*	0.005	1.012	1.003	1.022
Age square	−0.008**	0.004	0.992	0.985	0.999	−0.028+	0.010	0.972	0.955	0.989	−0.013*	0.005	0.987	0.977	0.996
Gender (reference: male)	−0.067***	0.016	0.935	0.906	0.966	−0.088***	0.016	0.916	0.887	0.946	−0.117***	0.017	0.889	0.861	0.919
**Marital status (reference: unmarried)**												
Married	0.023	0.032	1.024	0.962	1.089	0.061+	0.034	1.063	0.995	1.137	0.018	0.033	1.018	0.954	1.087
Divorced/Widowed	0.037^+^	1.038	1.038	0.950	1.134	0.084+	0.048	1.087	0.990	1.194	0.037	0.047	1.038	0.946	1.138
**Education status (reference: Primary school education)**												
Junior high school education	−0.221***	0.020	0.802	0.771	0.834	−0.199***	0.034	0.820	0.788	0.853	−0.224***	0.021	0.799	0.767	0.832
Higher school education	−0.469***	0.026	0.626	0.595	0.659	−0.433***	0.027	0.648	0.615	0.683	−0.454***	0.027	0.635	0.603	0.670
Associate Degree and above	−0.875***	0.030	0.417	0.393	0.442	−0.744***	0.031	0.475	0.448	0.505	−0.748***	0.031	0.473	0.445	0.503
Income	−0.005***	0.002	0.995	0.991	0.998	−0.009***	0.002	0.991	0.988	0.995	−0.014***	0.002	0.986	0.982	0.989
SRH	0.371***	0.017	1.449	1.400	1.499	0.308***	0.018	1.360	1.314	1.408	0.145***	0.019	1.156	1.115	1.199
Chronic disease	−0.536***	0.022	0.585	0.560	0.611	−0.547***	0.022	0.578	0.554	0.604	−0.670***	0.023	0.544	0.520	0.568
**Basic medical insurance (reference: None)**												
NRCMS	0.671***	0.025	1.956	1.862	2.054	0.725***	0.025	2.064	1.965	2.169	0.730***	0.026	2.075	1.973	2.181
URBMI	−0.512***	0.035	0.598	0.558	0.641	−0.493***	0.035	0.611	0.570	0.655	−0.482***	0.036	0.618	0.576	0.662
UEBMI	−0.717***	0.032	0.488	0.459	0.520	−0.694***	0.032	0.500	0.469	0.532	−0.663***	0.033	0.515	0.483	0.549
**Variance components**												
Period											0.390^+^	0.277			
Cohort						2.4190**	0.965				0.002^+^	0.001			
**Random effect**												
**Cohort**															
Pre-1935						2.653***	0.434	14.202	6.067	33.248	−0.015	0.040	0.996	0.927	1.071
1936–1940						2.032***	0.426	7.632	3.311	17.591	0.002	0.038	0.993	0.927	1.064
1941–1945						1.685***	0.422	5.390	2.358	12.320	0.015	0.034	1.017	0.955	1.082
1946–1950						1.270**	0.419	3.560	1.565	8.099	0.014	0.030	1.015	0.960	1.074
1951–1955						0.881*	0.418	2.414	1.064	5.477	0.028	0.027	1.022	0.971	1.076
1956–1960						0.466	0.418	1.593	0.702	3.613	−0.017	0.027	0.986	0.936	1.038
1961–1965						0.077	0.418	1.080	0.476	2.448	0.001	0.026	0.993	0.945	1.043
1966–1970						−0.251	0.418	0.778	0.343	1.764	0.008	0.025	1.007	0.960	1.057
1971–1975						−0.619	0.418	0.539	0.237	1.222	−0.012	0.026	0.991	0.944	1.040
1976–1980						−0.959*	0.419	0.384	0.169	0.871	0.004	0.027	1.004	0.954	1.056
1981–1985						−1.439***	0.419	0.237	0.104	0.539	−0.085**	0.027	0.920	0.874	0.969
1986–1990						−1.689***	0.421	0.185	0.081	0.421	−0.012	0.028	0.990	0.939	1.044
1991–1995						−1.923***	0.423	0.146	0.064	0.335	0.049	0.032	1.049	0.987	1.115
Post-1995						−2.185***	0.429	0.112	0.049	0.261	0.022	0.038	1.022	0.952	1.097
**Period**												
2010.000											1.067***	0.281	2.905	1.676	5.034
2012.000											−0.07	0.280	0.935	0.541	1.618
2014.000											−0.118	0.280	0.886	0.512	1.533
2016.000											−0.527*	0.280	0.592	0.342	1.025
2018.000											−0.352	0.280	0.701	0.405	1.213
**Model fit**												
−2RLPL	96208.49a					387014.900					389391.600				
*c* ^2^	81244.630					84924.410					84761.970				
*c*^2^/df	1.000					0.990					0.990				

+, ^*^, ^**^, ^***^ denotes significant at the significant at the significance level of 0.10, 0.05, 0.01, 0.001, respectively; the same as follow.

### The effect of basic medical insurance participation on the behavior of seeking primary care

Results in Table 1 show that after controlling for age, period and cohort variables, the preference for basic medical insurance participation was significantly associated with the selection of primary care seeking behaviors. Compared with the uninsured group, participants of NRCMS were more likely to seek primary care (OR = 2.075). On the contrary, participants of URBMI (OR = 0.618) and UEBMI (OR = 0.515) have a lower incidence of primary care.

### The effect of basic medical insurance participation varies with age, period, and cohort

#### The effect of basic medical insurance on primary care with age

Results in Table 2 show that the interaction between basic medical insurance participation and age was significant for URBMI (OR = 0.994) and UEBMI (OR = 0.994), but not significant for NRCMS. Compared with those who were not insured, the incidence of primary care for the 45.753 year old who participated in URBMI or UEBMI was lower (OR = 0.615, OR = 0.511, respectively). The interaction effects between URBMI, UEBMI became stronger, and the gap between in probability of primary care between those with and without basic insurance gradually widened, while the probability of primary care for the NRCMS groups was lower than that of non-insured groups. The probability of insured group at primary level varied non-significantly.

**Table 2:** The effect of basic medical insurance on behavior of seeking primary care with age.

**Model4 (*****N*** = **85,316)**					
	**Estimate**	**Standard error**	**OR**	**95%CI**	
				**Lower**	**Upper**
**Fixed effect**					
Intercept	0.816*	0.281	2.261	1.037	4.932
**Control variables**	-	-			
Age	0.012^+^	0.005	1.012	1.002	1.022
Age square	−0.012*	0.005	0.988	0.978	0.997
**Basic medical insurance (reference: None)**					
NRCMS	0.735***	0.026	2.085	1.982	2.194
URBMI	−0.486***	0.036	0.615	0.573	0.660
UEBMI	−0.671***	0.033	0.511	0.479	0.546
Age*Basic medical insurance (reference: Age*None)				
Age*NRCMS	0.003	0.002	1.003	0.999	1.006
Age*URBMI	−0.006^+^	0.002	0.994	0.990	0.999
Age*UEBMI	−0.006*	0.002	0.994	0.990	0.998
**Variance components**					
Period	0.385+	0.273			
Cohort	0.002+	0.001			
**Random effect**					
Period	-	-			
Cohort	-	-			
**Model fit**					
−2RLPL		389493.200			
*c* ^2^		84795.320			
*c*^2^/df		0.990			

+, *, ***denotes significant at the significant at the significance level of 0.10, 0.05, 0.001, respectively.

#### The effect of basic medical insurance on primary care with period

Results in Table 3 show that, the interaction items between the NRCMS and 2010 (OR = 1.340), 2018 (OR = −0.788) were significant. Compared with the uninsured population in 2010 and 2018, the probability of primary care was 3.490[≈exp(0.734+0.292)], 1.642[≈exp(0.734–0.238)], large in those insured population, suggesting a higher increase rate than the average.

**Table 3:** The effect of basic medical insurance participation on primary care with period.

**Model5 (*****N*** = **85,316)**					
	**Estimate**	**Standard error**	**OR**	**95%CI**	
				**Lower**	**Upper**
**Fixed effect**					
Intercept	0.917+	0.260	2.501	1.219	5.130
Control variables	-	-			
**Basic medical insurance (reference: None)**					
NRCMS	0.734***	0.010	2.084	1.677	2.589
URBMI	−0.542***	0.103	0.581	0.464	0.728
UEBMI	−0.751***	0.102	0.472	0.378	0.590
**Variance components**					
Period	0.302^+^	0.219			
Cohort	0.001*	0.001			
basic medical insurance *period	0.023^+^	0.010			
**Random effect**					
Period					
NRCMS*period2010	0.292*	0.102	1.340	1.096	1.637
NRCMS*period2018	−0.238*	0.097	0.788	0.651	0.954
Cohort	-	-			
**Model fit**					
−2RLPL		390795.100			
*c* ^2^		85305.240			
*c*^2^/df		1.000			

+, *, ***denotes significant at the significant at the significance level of 0.10, 0.05, 0.001, respectively.

#### The effect of basic medical insurance participation on primary care with cohort

Results in Table 4 showed that the interaction items between NRCMS and cohort pre1935, cohort 1986 and cohort 1991, and the interaction items between UEBMI and cohort 1991 were significant. Under the average cohort effect, in cohort pre1935, compared with the uninsured group, the rate of primary care to NRCMS group was 2.442 [≈exp(0.751+0.142)].

**Table 4:** The effect of basic medical insurance participation on variation of primary care with cohort.

**Model 6 (*****N*** = **81,276)**					
	**Estimate**	**Standard error**	**OR**	**95%CI**	
				**Lower**	**Upper**
**Fix effect**					
Intercept	0.851*	0.283	2.342	1.067	5.140
**Control variables**	-	-			
**Basic medical insurance (reference: None)**					
NRCMS	0.751***	0.046	2.118	1.930	2.325
URBMI	−0.473***	0.053	0.623	0.560	0.694
UEBMI	−0.655***	0.051	0.520	0.469	0.576
**Variance components**					
Period	0.384^+^	0.272			
Cohort	0.002	0.003			
Basic medical insurance *cohort	0.007**	0.003			
**Random effect**					
Period	**-**	-			
Cohort					
NRCMS*cohort pre1935	0.142^+^	0.074	1.153	0.997	1.333
NRCMS*cohort1986	−0.157**	0.052	0.855	0.772	0.947
NRCMS*cohort1991	−0.096^+^	0.057	0.909	0.813	1.015
UEBMI*cohort1991	0.156**	0.007	1.169	1.017	1.344
**Model fit**					
−2RLPL		389596.800			
*c* ^2^		84765.760			
*c*^2^/df		0.990			

+, *, **, ***denotes significant at the significant at the significance level of 0.10, 0.05, 0.01, 0.001, respectively.

**Figure 1:**
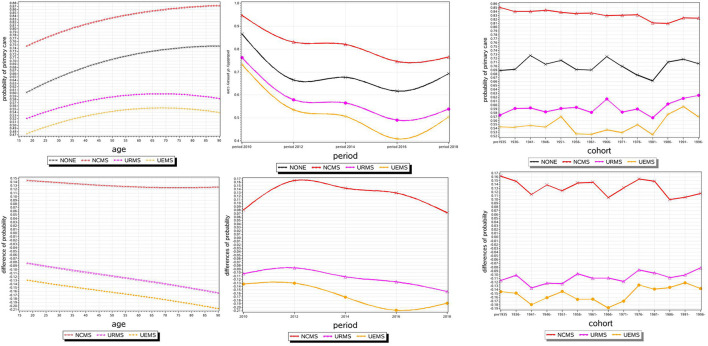
The effect of basic medical insurance participation on primary care with age, period, and cohort.

In the cohort 1986, compared with the uninsured group, the rate of primary care to NRCMS group was 1.811 [≈exp(0.751–0.157)]. In the 1991 cohort, compared with the uninsured group, the rate of primary care to NRCMS group was 1.925 [≈exp(0.751–0.096)], and the rate of primary care to the UEBMI group was 0.607 [≈exp(–0.656+0.156)]. The effect of basic medical insurance participation on primary care across successive cohorts as shown in Figure 1 [below]. Under different basic medical insurance participation types, the difference of in primary care in different cohorts was relatively small, especially in the NRCMS group. In addition, similar to the estimated results of Model 3, the 1981 cohort had the lowest probability of seeking primary care behavior in each insured group. Since then, as the birth cohort gets younger, the probability of consultation at the primary level has increased to varying degrees. In addition, we set up a full model considering age, period, cohort and medical insurance participation and interaction at the same time. The model results are shown in the Additional file 4: Supplementary Table 3.

## Discussion

The study analyzed the CFPS (2010, 2012, 2016 and 2018) data using the advanced HAPC-CCREM method. Findings of the study revealed the effect of basic medical insurance on the changes in primary care-seeking behavior. To the best of our knowledge, this is the first study using the HAPC-CCREM model to investigate the impact of basic changes in medical insurance on primary care. Findings of the study will provide evidence supporting policy formulation on the changes in basic medical insurance in primary care behavior.

First of all, study results indicated that the age effect curve was a univariate quadratic curve with an opening downward, showing an inverted U-shaped distribution, in line with the general life cycle research results on primary medical care behavior. Then, the period effect showed that the probability of primary care was the highest in 2010, and its probability declined since then, but rebounded in 2018, that may be attributed to the fact that since 2015, China has continuously promoted the construction of a hierarchical diagnosis and treatment system, and the service capabilities of primary medical institutions have been improved, and people were more willing to go to primary medical institutions for seeking medical treatment. Finally, only the cohort born in 1981–1985 had a significant impact on the probability of primary care. This may be related to the distrust of young people in the service capabilities of primary medical institutions.

One of the main findings of the study was that the participation of basic medical insurance had significant effect on residents went to primary medical facilities treatment. The NRCMS was increased the possibility of treatment at the primary level, which is consistent with the previous studies (17–24). The participation of UEBMI and URBMI both had negative effect on residents' primary medical care, consistent with previous studies (25, 26). The differences in the direction of effect of NRCMS, URBMI and UEBMI on primary care may be attributable to the rural-urban differences. For example, the people covered by NRCMS are mainly rural residents, and it is more inconvenient for them to visit high-level hospitals, compared to their urban counterparts who were covered by URBMI and UEBMI. Instead, benefit from the convenience of transportation, rural residents are more likely to visit the primary medical institutions for medical treatment (27). On the other hand, it is easier for urban patients to visit the secondary and tertiary healthcare institutions, which are usually located in urban areas (28, 29). Thus, they are more likely to bypass primary medical institutions and choose these higher-level facilities, regardless of the severity of the disease (30–32). However, the quality of rural medical services was poor (33), underscoring the urgent need of strengthening the quality of primary medical services in rural areas in China.

With the increase of age, the probability of primary care changed in an inverted “U” shape. The effect of age increase on NRCMS is positive, but it is small and not statistically significant. The increase of age makes the participation of UEBMI and URBMI to increase the negative impact on primary care. The gap of primary care probability between UEBMI and URBMI insured groups and the non-insured groups gradually widens. The potential reasons were that people's physical condition deteriorates due to age, and the demand for health services inhibits the effectiveness of differentiated reimbursement methods. Urban residents living in urban areas and UEBMI insured residents are more willing to go directly to high-level hospitals to receive higher level of medical services.

From 2010 to 2016, with the change of period, the probability of primary care declined, but in 2018 there was a rebound. This benefited from China's efforts in strengthening the construction of primary medical care in recent years, and it also showed that the construction of China's hierarchical diagnosis system has played a certain role in guiding residents to the primary for medical treatment (34, 35). In the study of the impact of basic medical insurance participation on the probability of primary care over time, the results showed that the positive impact of the NRCMS on the probability of primary care was strongest in 2010 and weakest in 2018. However, in the study of basic medical insurance on the probabilities of primary care, the effect of URBMI and UEBMI on the probability of primary care was not statistically significant. As for NRCMS participation, its influence on the probability of primary care showed a trend of first increasing and then decreasing with the passage of the period. The agricultural reform in 1978 made China's 900 million farmers lose medical insurance. With the transformation of the collective economic system in the early 1980s, the coverage rate dropped to 5% in 1985.Since >80% of the rural residents were not covered by any medical insurance before the introduction of the NRCMS (36), in 2003 only 9.5% of rural residents were covered by Cooperative Medical System (37). From 1985 to 2003, rural residents in China were basically in a vacuum of medical insurance. The promulgation of the NRCMS in 2003 and the period from 2009 to 2011 were the first phases of China's new medical reform, emphasizing on expanding medical insurance participation, strengthening infrastructure construction, and improving medical utilization (35). The research data from CHARLS have shown that there were still many shortcomings in the combination of China's NRCMS and medical services, but its convenience and price advantages enhance the promotion of basic medical insurance at the primary level. With the increasing demand for people's health, motorized travel has ensured the accessibility of high-level medical and health services, and the overall reimbursement rate of the NRCMS continues to increase. Accessibility and affordability are further improved, at this time the positive impact of basic medical insurance on primary care is suppressed (38).

The effect of the cohort on the probability of primary care passed the statistical test at a lower level of significance, and the degree of influence was weak. Different birth cohort groups had relatively small fluctuations in the probability of seeking medical treatment at primary level. Individuals born between 1981 and 1985 had the lowest probability of primary care. This was slightly inconsistent with the life cycle theory, but it was very suitable to be explained by the idea of recency effect. Compared with people's past historical experience, the current reality was more likely to influence people's consciousness and behavior. Although the population of different birth cohorts had undergone different historical changes, the current reality was more effective in influencing the impact of residents on primary care.

The above findings have some implications for medical reform in China. In order to effectively promote China's medical reform, the Chinese government can promote residents' choice of primary care by increasing the gap in the reimbursement ratio of medical insurance and publicizing the high reimbursement ratio for grass-roots treatment. In addition, our results to some extent hint at the importance and necessity of allocating primary health care resources, especially in rural areas. The Chinese government should further balance the allocation of healthcare resources and enhance the capacity of primary health care services.

This study has several limitations. First, the overall level of medical insurance is low, and its implementation in different regions is slightly different. Secondly, the measure of primary care-seeking behavior was incomprehensive. Primary care-seeking behavior could also include choosing between home remedies, pharmacies and stores, or indigenous healers and doctors. However, due to limitations in the data, the current study includes only inpatient treatment, outpatient treatment, and physical examinations. To provide a deeper understanding of primary care-seeking behavior, further efforts incorporating other aspects of primary care-seeking behavior are necessary. Finally, survey data were based entirely on self-reports and thus may be subject to recall bias. Patients' perceived experiences may vary as a result of their expectations and unique characteristics.

## Conclusions

Findings of the study showed that the participation of Chinese residents in basic medical insurance during 2010-2018 will indeed affect the behavior of seeking primary care with age, period and cohort. We have observed that with the changes of age, period and cohort, age and period showed a more significant regulating effect on primary care. The study of primary care seeking behavior is an important direction for the construction of a graded diagnosis system. Basic medical insurance is the starting point of the graded diagnosis system. We can provide some guidance for policy formulation on the changes in basic medical insurance in the basic medical treatment behavior.

## Data availability statement

The original contributions presented in the study are included in the article/Supplementary material, further inquiries can be directed to the corresponding author/s.

## Author contributions

Methodology: NC. Supervision: WX. Writing—original draft: XL. Writing—review and editing: NC, XL, JJ, and WX. All authors contributed to the article and approved the submitted version.

## Conflict of interest

The authors declare that the research was conducted in the absence of any commercial or financial relationships that could be construed as a potential conflict of interest.

## Publisher's note

All claims expressed in this article are solely those of the authors and do not necessarily represent those of their affiliated organizations, or those of the publisher, the editors and the reviewers. Any product that may be evaluated in this article, or claim that may be made by its manufacturer, is not guaranteed or endorsed by the publisher.

## References

[1] China. NHaFPCo. China Health Statistical Yearbook 2015. Beijing: Peking Union Medical College Press (2016).

[2] National Health Commission of the People's Republic of China. Statistical Bulletin on the Development of China's Health Care. Beijing (2019).

[3] Council TS. Graded Diagnosis and Treatment Pilot Work Evaluation Criteria. Beijing: The State Council (2015).

[4] Zhang ANZ Mossialos E. Does health insurance reduce out-of-pocket expenditure? Heterogeneity among China's middle-aged and elderly. Soc Sci Med. (2017) 190:11–9. 10.1016/j.socscimed.2017.08.005 28823943

[5] Yang Y. Age, Period, Cohort Effects. In*: Deborah Carr (Ed) Encyclopedia of the Life Course and Human Development*. Detroit: Macmillan Reference USA (2008) 3:6–10.

[6] Alemayehu B Warner KE. The lifetime distribution of health care costs. Health Serv Res. (2004) 39:627–42. 10.1111/j.1475-6773.2004.00248.x 15149482 PMC1361028

[7] Li J Feng X. Status and influencing factors of hierarchical medical system among middle -aged and elderly patients in China. Chin Gen Pract. (2017) 20:2316–23. 10.3969/j.issn.1007-9572.2017.19.005

[8] Wong WCW Jiang SF Ong JJ Peng MH Wan E Zhu SZ. Bridging the gaps between patients and primary care in china: a nationwide representative survey. Ann Fam Med. (2017) 15:237–45. 10.1370/afm.2034 28483889 PMC5422085

[9] Yu WY Li MN Ye F Xue C Zhang LL. Patient preference and choice of healthcare providers in Shanghai, China: a cross-sectional study. Bmj Open. (2017) 7:6418. 10.1136/bmjopen-2017-016418 29092898 PMC5695435

[10] Xie Y Hu JW. an introduction to the China family panel studies (CFPS). Chinese Sociol Rev. (2014) 47:3–29.

[11] Minhas ACV Sharma S. Health care seeking behavior of parents of under five in District Kanga, Himachal Pradesh. Int J Commun Med Public Health. (2018) 5:229. 10.18203/2394-6040.ijcmph20180229

[12] Zhou Z Zhao Y Shen C Lai S Nawaz R Gao J. Evaluating the effect of hierarchical medical system on health seeking behavior: a difference-in-differences analysis in China. Soc Sci Med. (2021) 268:113372. 10.1016/j.socscimed.2020.113372 32979776

[13] Guo JW Wu Q. Does basic medical insurance promote health and subjective fairness among residents. Soc Sec Stud. (2021) 01:1–11. 10.3969/j.issn.1674-4802.2021.03.006

[14] Yang Y Land KC. A mixed models approach to the age-period-cohort analysis of repeated cross-section surveys, with an application to data on trends in verbal test scores. Sociol Methodol. (2006) 36:75–97. 10.1111/j.1467-9531.2006.00175.x

[15] Frenk SM Yang YC Land KC. Assessing the significance of cohort and period effects in hierarchical age-period-cohort models: applications to verbal test scores and voter turnout in U. S presidential elections. Soc Forces. (2013) 92:221–48. 10.1093/sf/sot066 25392566 PMC4226416

[16] Rumberger RW. Hierarchical linear models: Applications and data analysis methods - Bryk, AS, Raudenbush, SW. Econ Educ Rev. (1997) 16:348. 10.1016/S0272-7757(97)80188-4

[17] Wagstaf A Lindelow M. Jun G. Extending health insurance to the rural population: an impact evaluation of China's new cooperative medical scheme. J Health Econ. (2009) 28:1–19. 10.1016/j.jhealeco.2008.10.007 19058865

[18] Liu D Tsegai D. The new cooperative medical scheme and its implications for access to health care and medical expenditure: evidence from rural China. Center Develop Res. (2011) 155:23. 10.22004/AG.ECON.116746

[19] Brown PH Theoharides C. Health-seeking behavior and hospital choice in China's new cooperative medical system. Health Econ. (2009) 18:S47–64. 10.1002/hec.1508 19551751

[20] Babiarz KS Miller G Yi H. New evidence on the impact of China's New Rural Cooperative Medical Scheme and its implications for rural primary healthcare: multivariate difference-in-difference analysis. Br Med J. (2010) 341:c5617. 10.1136/bmj.c5617 20966008 PMC6173169

[21] Babiarz KS Miller G Yi H. China's new cooperative medical scheme improved finances of township health centers but not the number of patients served. Health Affairs. (2012) 31:1065–74. 10.1377/hlthaff.2010.1311 22566448

[22] Dai B. Zhou, J, Mei, YJ. Can the New Cooperative Medical Scheme promote rural elders' access to healthcare services? Geriat Gerontol Int. (2011) 11:239–45. 10.1111/j.1447-0594.2011.00702.x 21545383

[23] Hou Z Van de. Poel, E, Van Doorslaer, E. Effects of NCMS on access to care and financial protection in China. Health Economics. (2014) 23:917–34. 10.1002/hec.2965 23983020

[24] Sun LS. Does health insurance lead to improvement of health status among chinese rural adults? Evidence from the China family panel studies. Int J Health Serv. (2020) 50:350–9. 10.1177/0020731420914824 32517568

[25] Zhang A NZ Albala S A. Patient choice of health care providers in china: primary care facilities vs. hospitals. Health Syst Reform. (2020) 6:e1846844. 10.1080/23288604.2020.1846844 33314985

[26] Zheng L. Has health insurance system changed health care seeking behavior? –Evidence from Chinese CHNS. Public Finance Res. (2017) 02:84–97.

[27] Liu Y Zhong L Yuan S van de Klundert J. Why patients prefer high-level healthcare facilities: a qualitative study using focus groups in rural and urban China. BMJ global health. (2018) 3:e000854. 10.1136/bmjgh-2018-000854 30258653 PMC6150133

[28] Chen Y Yin Z Xie Q. Suggestions to ameliorate the inequity in urban/rural allocation of healthcare resources in China. Int J Equity Health. (2014) 13:34. 10.1186/1475-9276-13-34 24884614 PMC4016733

[29] Cai M Liu EC Li W. Rural vs. urban patients: benchmarking the outcomes of patients with acute myocardial infarction in Shanxi, China from 2013 to 2017. Int J Env Res Pub He. (2018) 15:1930. 10.3390/ijerph15091930 30189602 PMC6165441

[30] Yip W HW. Harnessing the privatisation of China's fragmented health-care delivery. Lancet. (2014) 384:805–18. 10.1016/S0140-6736(14)61120-X 25176551 PMC7159287

[31] Wu D Lam TP. Underuse of primary care in china: the scale, causes, and solutions. J Am Board Fam Med. (2016) 29:240–7. 10.3122/jabfm.2016.02.150159 26957381

[32] Li X Lu J Hu S Cheng KK De Maeseneer J Meng Q. The primary health-care system in China. Lancet. (2017) 390:2584–94. 10.1016/S0140-6736(17)33109-4 29231837

[33] Shi YJ Yi HM Zhou H Zhou CC Xue H Rozelle S. The quality of primary care and correlates among grassroots providers in rural China: a cross-sectional standardized patient study. Lancet. (2017) 390:S16–S. 10.1016/S0140-6736(17)33154-9

[34] Ma X WH Yang L. Realigning the incentive system for China's primary healthcare providers. Br Med J. (2019) 365:l2406. 10.1136/bmj.l2406 31227522 PMC6598721

[35] Yip W Fu H Chen AT Zhai T Jian W Xu R. 10 years of health-care reform in China: progress and gaps in Universal health coverage. Lancet. (2019) 394:1192–204. 10.1016/S0140-6736(19)32136-1 31571602

[36] Liu X. Cao, H. China's cooperative medical system: Its historical transformations and the trend of development. J Public Health Pol. (1992) 13:501–11. 10.2307/3342538 1287043

[37] Health Mo. Reports on the 2003 National Health Services Survey Results. Technical Report Center for Statistics Information, Ministry of Health, Chin. (2004).

[38] Dai T. The construction, achievements and prospect of health system in China. Chinese *J Health Pol*. (2019) 12:1–7. 10.3969/j.issn.1674-2982.2019.10.001

