# Supplementary material for: The effect of basic medical insurance on the changes of primary care seeking behavior: An application of hierarchical age-period-cohort analysis
Source: Front Public Health. 2022 Aug 3;10:929896. doi: 10.3389/fpubh.2022.929896 (PMC9382404; doi:10.3389/fpubh.2022.929896)
Supplement: Supplementary file 1 [file Table_1.docx]

***Supplementary Material***

1. **Supplementary Tables and Figures**

## 1.1 Supplementary Tables

| Supplementary Table 1. The distribution of behavior of seeking primary care among groups with different characteristics | | | | | | | | | | |
| --- | --- | --- | --- | --- | --- | --- | --- | --- | --- | --- |
| Variables | | Total residents（N=85316） | | | Primary care=0  （N=27170） | | | Primary care=1  （N=58146） | | |
|  |  | N/Mean* | | %/SD* | N/Mean* | | %/SD* | N/Mean* | | %/SD* |
| Age | | | | | | | | | | |
|  | Mean (SD) | 45.74 (15.06) |  | | 44.51 | 15.06 | | 46.31 | 15.02 | |
| Gender | | | | | | | | | | |
|  | Female | 42084 | 49.33 | | 13447 | 15.76 | | 28637 | 33.57 | |
|  | Male | 43232 | 50.67 | | 13723 | 16.08 | | 29509 | 34.59 | |
| Marital status | | | | | | | | | | |
|  | Unmarried | 8551 | 10.02 | | 3267 | 12.02 | | 5284 | 9.08 | |
|  | Married | 71348 | 83.63 | | 22271 | 81.97 | | 49077 | 84.40 | |
|  | Divorced/Widowed | 5417 | 6.35 | | 1632 | 6.00 | | 3785 | 6.51 | |
| Education status | | | | | | | | | | |
|  | Primary school | 41854 | 49.06 | | 9966 | 36.68 | | 31888 | 54.84 | |
|  | Junior high school | 23850 | 27.95 | | 7320 | 26.94 | | 16530 | 28.43 | |
|  | Higher school | 10515 | 12.32 | | 4518 | 16.63 | | 5997 | 10.31 | |
|  | Associate Degree or above | 9097 | 10.66 | | 5366 | 19.75 | | 3731 | 6.42 | |
| Income | | | | | | | | | | |
|  | Mean (SD) | 12504.39 | 120889 | | 18136 | 17560 | | 9532 | 15650 | |
| SRH | | | | | | | | | | |
|  | unhealth | 54275 | 63.62 | | 18889 | 69.52 | | 35386 | 60.86 | |
|  | health | 31041 | 36.38 | | 8281 | 30.48 | | 22760 | 39.14 | |
| Chronic disease | | | | | | | | | | |
|  | no | 70000 | 86.13 | | 21393 | 26.32 | | 48607 | 59.8 | |
|  | yes | 11276 | 13.87 | | 4718 | 5.8 | | 6558 | 8.07 | |
| Basic medical insurance | | | | | | | | | | |
|  | none | 8251 | 10.11 | | 3203 | 3.94 | | 5012 | 6.17 | |
|  | NRCMS | 56755 | 69.83 | | 13104 | 16.12 | | 43651 | 53.71 | |
|  | URBMI | 5761 | 7.09 | | 3118 | 3.84 | | 2643 | 3.25 | |
|  | UEBMI | 10545 | 12.97 | | 6686 | 8.23 | | 3895 | 4.75 | |
| Note: *Mean and SD were performed for continuous variables, N and % were performed for categorical variables. | | | | | | | | | | |

| Supplementary Table 2. The prevalence of behavior of seeking primary care in China by period, cohort | | | | | | |
| --- | --- | --- | --- | --- | --- | --- |
|  | 2010 | 2012 | 2014 | 2016 | 2018 |  |
| pre1935 | 0.827(231) | 0.610(451) | 0.690(29) | 0.833(12) | 0.625(16) |  |
| cohort 1936 | 0.888(277) | 0.639(646) | 0.760(121) | 0.750(48) | 0.679(106) |  |
| cohort 1941 | 0.891(469) | 0.687(1135) | 0.791(363) | 0.617(128) | 0.650(406) |  |
| cohort 1946 | 0.871(684) | 0.673(1876) | 0.765(767) | 0.696(224) | 0.691(960) |  |
| cohort 1951 | 0.887(967) | 0.714(2712) | 0.745(1442) | 0.682(377) | 0.659(1712) |  |
| cohort 1956 | 0.896(768) | 0.680(2413) | 0.702(1481) | 0.593(403) | 0.623(1702) |  |
| cohort 1961 | 0.905(1104) | 0.694(3106) | 0.705(2210) | 0.612(659) | 0.628(2526) |  |
| cohort 1966 | 0.902(1176) | 0.726(3308) | 0.707(2571) | 0.634(702) | 0.636(2920) |  |
| cohort 1971 | 0.897(962) | 0.691(2816) | 0.673(2171) | 0.612(689) | 0.637(2471) |  |
| cohort 1976 | 0.839(739) | 0.685(2096) | 0.652(1719) | 0.620(606) | 0.599(1893) |  |
| cohort 1981 | 0.828(575) | 0.646(2022) | 0.593(1688) | 0.553(687) | 0.564(2042) |  |
| cohort 1986 | 0.847(549) | 0.668(2207) | 0.593(2069) | 0.567(1230) | 0.559(2656) |  |
| cohort 1991 | 0.840(131) | 0.725(1413) | 0.654(986) | 0.582(839) | 0.557(1822) |  |
| cohort 1996 | (0) | 0.333(3) | 0.700(100) | 0.635(197) | 0.596(690) |  |

| Supplementary Table 3. The effect of basic medical insurance participation on variation of primary care with age, period, cohort | | | | | |
| --- | --- | --- | --- | --- | --- |
|  | Model7(N =85316) | | | | |
|  | Estimate | Standard Error | OR | 95%CI | |
|  |  |  |  | Lower | Upper |
| **Fix Effect** | |  |  |  |  |
| Intercept | 0.887* | 0.261 | 2.428 | 1.176 | 5.012 |
| Control variables | |  |  |  |  |
| Age | -0.001^+^ | 0.001 | 1.008 | 0.997 | 1.019 |
| Age square | -0.118*** | 0.017 | 1.000 | 1.000 | 1.000 |
| Basic medical insurance (reference: None) | | |  |  |  |
| NRCMS | 0.735*** | 0.046 | 2.085 | 1.679 | 2.591 |
| URBMI | -0.534*** | 0.053 | 0.586 | 0.468 | 0.734 |
| UEBMI | -0.751*** | 0.102 | 0.472 | 0.378 | 0.590 |
| Age*Basic medical insurance(reference: Age*None) | | |  |  |  |
| Age*NRCMS | 0.001 | 0.002 | 1.001 | 0.998 | 1.005 |
| Age*URBMI | -0.006** | 0.003 | 0.994 | 0.989 | 0.999 |
| Age*UEBMI | -0.006*** | 0.002 | 0.994 | 0.989 | 0.999 |
| **Variance Components** | | |  |  |  |
| period | 0.308 ^+^ | 0.222 |  |  |  |
| cohort | 0.002 | 0.002 |  |  |  |
| basic medical insurance *period | 0.021* | 0.002 |  |  |  |
| basic medical insurance *cohort | 0.003^+^ | 0.003 |  |  |  |
| **Random Effect** | |  |  |  |  |
| period | **-** | - |  |  |  |
| NRCMS* period 2010 | 0.280** | 0.1 | 1.323 | 1.086 | 1.610 |
| NRCMS* period 2018 | -0.2334** | 0.1 | 0.792 | 0.657 | 0.954 |
| Cohort | - | - |  |  |  |
| **model fit** | |  |  |  |  |
| -2RLPL | 390905.1 | | | | |
| c^2^ | 85279.74 | | | | |
| c^2^/df | 1 | | | | |

+,*,**,***denotes significant at the significant at the significance level of 0.10,0.05,0.01,0.001,respectively.

**1.2 Supplementary Figures**


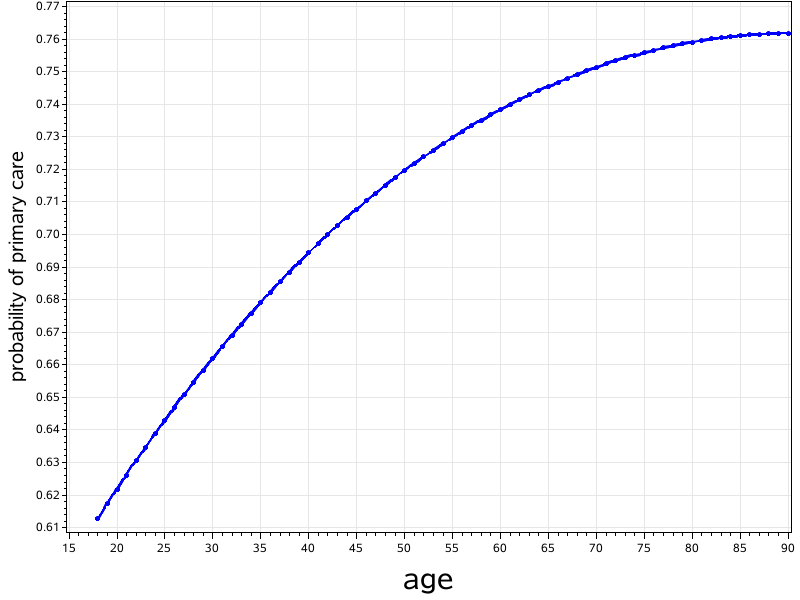

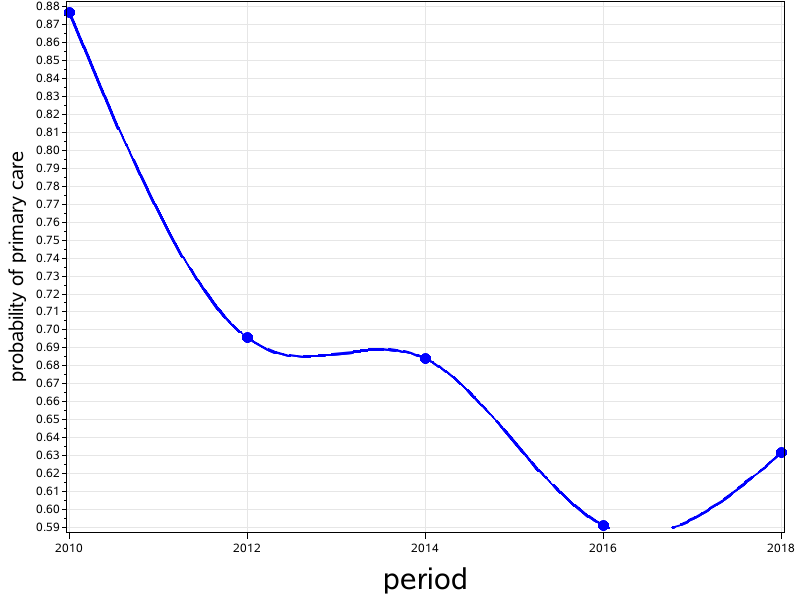

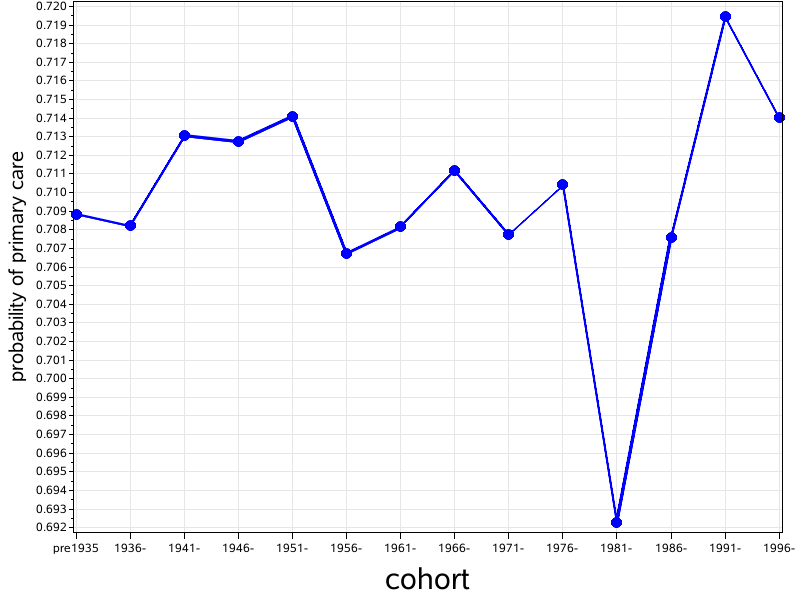


**Supplementary Figure 1.** The effect of seeking primary care behavior with age, period, and cohort
